# Online Concerns of Parents Suspecting Autism Spectrum Disorder in Their Child: Content Analysis of Signs and Automated Prediction of Risk

**DOI:** 10.2196/jmir.5439

**Published:** 2016-11-22

**Authors:** Ayelet Ben-Sasson, Elad Yom-Tov

**Affiliations:** ^1^University of HaifaHaifaIsrael; ^2^Microsoft Research & DevelopmentHerzeliaIsrael

**Keywords:** online queries, autistic disorders, parents, machine learning, early detection

## Abstract

**Background:**

Online communities are used as platforms by parents to verify developmental and health concerns related to their child. The increasing public awareness of autism spectrum disorders (ASD) leads more parents to suspect ASD in their child. Early identification of ASD is important for early intervention.

**Objective:**

To characterize the symptoms mentioned in online queries posed by parents who suspect that their child might have ASD and determine whether they are age-specific. To test the efficacy of machine learning tools in classifying the child’s risk of ASD based on the parent’s narrative.

**Methods:**

To this end, we analyzed online queries posed by parents who were concerned that their child might have ASD and categorized the warning signs they mentioned according to ASD-specific and non-ASD–specific domains. We then used the data to test the efficacy with which a trained machine learning tool classified the degree of ASD risk. Yahoo Answers, a social site for posting queries and finding answers, was mined for queries of parents asking the community whether their child has ASD. A total of 195 queries were sampled for this study (mean child age=38.0 months; 84.7% [160/189] boys). Content text analysis of the queries aimed to categorize the types of symptoms described and obtain clinical judgment of the child’s ASD-risk level.

**Results:**

Concerns related to repetitive and restricted behaviors and interests (RRBI) were the most prevalent (75.4%, 147/195), followed by concerns related to language (61.5%, 120/195) and emotional markers (50.3%, 98/195). Of the 195 queries, 18.5% (36/195) were rated by clinical experts as low-risk, 30.8% (60/195) as medium-risk, and 50.8% (99/195) as high-risk. Risk groups differed significantly (*P*<.001) in the rate of concerns in the language, social, communication, and RRBI domains. When testing whether an automatic classifier (decision tree) could predict if a query was medium- or high-risk based on the text of the query and the coded symptoms, performance reached an area under the receiver operating curve (ROC) curve of 0.67 (CI 95% 0.50-0.78), whereas predicting from the text and the coded signs resulted in an area under the curve of 0.82 (0.80-0.86).

**Conclusions:**

Findings call for health care providers to closely listen to parental ASD-related concerns, as recommended by screening guidelines. They also demonstrate the need for Internet-based screening systems that utilize parents’ narratives using a decision tree questioning method.

## Introduction

The increasing rate of diagnosed autism spectrum disorders (ASD) [1] along with the public’s growing awareness of the disorder leads more parents to suspect ASD in their child. These early concerns can arise months before parents decide to approach a professional [2]. As the majority of parents of children with ASD report having symptom-related concerns before the child reaches the age of 3 years [3-5], it stands to reason that reporting these concerns could potentially facilitate earlier evaluation and services. Parents of infants and toddlers seek information about their child’s development on online community forums, where they expect to be able to verify or discuss their concerns [6-8]. However, as the resources of health care professionals are scarce, machine learning tools could be applied to perform prescreening of parental forums where ASD concerns are voiced.

On the one hand, parents’ online descriptions of their concerns regarding their child's development offer the opportunity to facilitate early referrals; whereas on the other hand, the use of a free-text format makes it difficult to determine the degree of risk for a specific diagnosis. From a public health perspective, online queries are a window into the behaviors that parents recognize as alarming signs of ASD. In turn, this information can be used to design screening procedures to help parents detect signs that are not readily noticeable. Moreover, machine learning tools may offer a way to estimate the degree of ASD risk of the child whose symptoms were described in the online query. The goals of the study were two-fold: (1) To characterize the symptoms mentioned in online queries posed by parents who suspected that their child might have ASD and categorize queries according to the level of clinical risk and the age of the child; and (2) to test the efficacy of machine learning tools in classifying the child’s risk of ASD based on the parent’s narrative.

The entire process from the time the parents first suspect developmental problems until an ASD diagnosis is obtained can take years [2,9,10]. Studies have demonstrated that the time between parents’ first concerns and the first professional ASD consultation was between 5 and 8 months on average [2,9,10], and the average time from concern to diagnosis was more than 32 months [2,11]. ASD is behaviorally diagnosed, based on Diagnostic and Statistical Manual of Mental Disorders (DSM-V) [12] criteria describing symptoms in the domains of social-communication and repetitive and restricted behaviors and interests (RRBI). Of all neurodevelopmental disorders, early identification of ASD is particularly difficult, for the following reasons: (1) the range of what is considered typical, healthy social-communication development is very wide; (2) certain ASD symptoms such as language patterns cannot be detected and evaluated before the child reaches the age of 2 years; (3) diagnosis depends on clinical expertise rather than on a biological marker; (4) early signs of ASD and other neurodevelopmental disorders partly overlap leading to complex diagnostic issues; and (5) ASD screening tools show moderate sensitivity and specificity. Consequently, parents are often left to cope with their worries, long before a diagnosis can be made, which in turn increases their urge to approach online communities. The long-term goal of this study was to devise a computerized tool based on the parents’ narrative of concerns that could estimate their child’s ASD risk.

An algorithm for systematic screening of ASD, devised by the American Association of Pediatrics, is used by health care professionals to elicit parental concerns regarding their child’s development to calculate a cumulative ASD-risk score and conduct closer monitoring of children who were found to be at risk [13]. Thus, parents’ early concerns, which have been validated against ASD screening tools and subsequent diagnosis [14,15], constitute a source of valuable clinical information and often initiate the diagnostic process. Parental concerns are relatively easy to elicit, time-efficient, and bypass the challenges inherent in direct testing, which does not always reflect the child’s true skill level. Notwithstanding these advantages, the widespread concerns of parents of young children pose a challenge for the differential prediction of ASD. In addition, parents may be biased in identifying certain signs, due to their emotional state, beliefs, and developmental knowledge. The validity of parental concerns is a subject of continuous research, and has implications for the clinical interpretation of parental concerns.

Evidence shows that the number and type of early parental concerns are predictive of a later developmental disorder [16] and specifically of ASD [15-18]. Types of concerns are classified as ASD-specific (ie, related to core ASD symptoms) and non-ASD–specific concerns. Research has shown that both types of concerns are associated with an eventual ASD diagnosis (reported in more than 18% of cases). Predictive ASD-specific concerns related to communication, language, social-emotional responses, and stereotyped behaviors; whereas non-ASD–specific concerns related to behavior or temperament, regression of skills, medical problems, or delay in milestones [9,10,15]. Studies of siblings of children with ASD indicate that the most frequent or first concern of parents involves communication development [9,15,19-21]. In a large retrospective study of parental concerns in ASD, social-emotional concerns (including nonverbal communication) were the most prevalent, followed by language and RRBIs; however, non-ASD–specific concerns were mentioned as well by at least half of the sample [10]. Retrospective parent reports of motor problems, unusual sensory and repetitive behaviors, atypical play patterns, and behavioral problems differentiated children later diagnosed with ASD from those diagnosed with other developmental disabilities [22]. However, in another study [21], parental concerns unique for children with ASD were challenging behaviors and attention problems. Parental concerns that led to a differential diagnosis of atypical development (vs ASD) were related to motor and communication problems. Looking at the presence of a combination of concerns showed that parental concerns about behavioral problems and about cognitive delay in the absence of concerns about communication were not likely predictors of an ASD diagnosis [23]. This evidence underscores the need for an automated system, which can capture the combination of concerns in risk determination. This study analyzed the ASD- and non-ASD–specific signs reported online by parents who suspected ASD warning signs in their child’s development.

Some studies associate the age of the child when the parents’ concerns are first aroused with the type of signs and their predictive validity. Studies describing parental concerns in families at high risk for an ASD diagnosis concluded that the number of ASD-specific signs noted by concerned parents of 12-month-old children is a better predictor than the number of signs noted by concerned parents of 6-month-old children [15]. Research indicates that although communication was the most frequent first concern, it served to differentiate children with ASD from those with other disorders only among 24-month-old children [3]. Evidence shows that a second type of parental concern expressed frequently is in the behavior or temperament domains at 14 and 24 months and both behavior or temperament and social development domains at 36 months. This differentiation at 24 months and not earlier is in line with another study [17]. Non-ASD–specific concerns (in 53% of children) related to motor problems, anxiety, tantrums, and hyperactivity were associated with earlier parental awareness, whereas ASD-specific concerns, including social withdrawal, abnormal gaze, and poor social interaction, were associated with later parental concerns [10]. In this study, the types of concerns that alert parents of ASD were compared among different child age groups.

To summarize, the reviewed evidence supports the working hypothesis that early parental concerns from the child’s second year of life predict later ASD diagnosis. However, most of the studies were based on retrospective reports of parents with children with ASD [10,24], or prospective reports in a high-risk sample [3,15,17,19,22,25]. In high-risk samples, parental concerns represent cases in which there is a truly elevated likelihood of ASD in a younger sibling, and they are already on alert for specific signs [26]. Previous evidence relied on parental responses to structured questions about early concerns in specific areas, sometimes followed by a textual description of the concern mentioned [21]. The Internet reflects the distribution of spontaneous parental ASD concerns in the general population, thus providing access to the data long before they are reported to a professional. The availability of such data makes it possible to explore the potential construction of an automated risk indicator, based on free-text descriptions.

There are several projects that use technology to enable automated early identification of developmental problems, including ASD. For example, the Modified-Checklist for Autism in Toddlers-Revised (M-CHAT-R/F), comprising an ASD screening questionnaire and follow-up interview, has been implemented electronically. The M-CHAT-R/F has proved efficient in lowering both false-positives and negatives compared with paper-format screening [27]. In another study, the online Ages and Stages Questionnaire screener was comparable with the paper version [28]. These studies support the reliance on an Internet-based screening platform, but they do not look at free-text analysis of parental concerns emerging prior to engaging in an ASD-specific screening process.

Regardless of ASD, parents, especially of young first-borns, frequently seek health- and developmental-related information online [6,29]. The Internet was identified as parents’ third routine source for obtaining health information [6]. The Internet offers an anonymous round-the-clock platform for expressing concerns. The increased trend in parents’ online information seeking is related to sociodemographic changes, such as living at a geographical distance from parents, decrease in support from family and friends, information from the previous generation is perceived outdated, and there is a greater demand for experience-based information [8]. The clinical quality of answers provided online to parents suspecting ASD in their child varies greatly [30]. There is a need to develop online systems to support parents in interpreting their young child’s behavior, so as to validate concerns and offer further guidance to parents when appropriate, while also minimizing the risks of acting solely on nonprofessional online advice.

Machine learning tools [31] have been previously applied for predicting health-related conditions from text, but never for predicting ASD. We note that online forums pose a challenge for analysis, given their unstructured nature and users’ descriptions of their symptoms in nonmedical terms. De Choudhury et al [32] investigated the ability to detect clinical depression from social media postings. Search-engines queries were used to predict mood disorder episodes [33]. In another study, adolescents at risk of being bullied were identified using online texts from MTV’s A Thin Line project. Thousands of teenagers’ online posts were used to develop an algorithm for identifying offensive cyber bullying, based on topic modeling methods [34]. This study built upon these works to test the possibility of devising an automated ASD-risk estimator online, based on large amounts of unstructured textual data and a machine learning algorithm.

The remainder of the paper is organized as follows. The Methods section describes the nature of the sampled queries, coding procedures, and data analysis steps. The Results section describes the types of signs mentioned by parents and their comparison between ASD risk groups and age groups. This section ends with the description of automated prediction of ASD risk from the text. The Discussion section lays out the interpretations and implications of the study.

## Methods

### Sample

This study utilized the Yahoo Answers platform to examine queries of parents suspecting their child has ASD. On the Yahoo Answers platform, queries are posted using natural language, answers are submitted by users, and a community forms around this interaction. A query can elicit multiple answers; one of them is rated the best answer, either by the asker or by the community. Yahoo Answers queries—rather than search-engine queries—were selected for the purpose of this study, as they consist of anonymously posted queries on a public platform and hence are more likely to represent parents’ true need for an answer.

We extracted all English-language queries from Yahoo Answers that were submitted between 6 June, 2006 and 12 December, 2013 and contained the words *autism*, *Asperger*, *ASD*, or *PDD*. A total of 8681 queries met these criteria. We used crowdsourcing [35] (using CrowdFlower) to differentiate between queries posted by parents of a child diagnosed with autism (n=2412), queries posted by parents of a child diagnosed with autism whose sibling they suspected might have autism (n=41), queries posted by parents who suspected their child might have autism (n=1081), and queries posted by parents who did not match any of the above descriptions (n=5147).

Of the 1081 queries of suspecting parents, 195 were randomly selected and analyzed for this study. Among these, in 96.4% (188/195) of the queries that mentioned the age of the child, it was, on average, 3.2 years (SD 2.9; range 1.25 months-18 years; 60.3% [114/189] below 3 years of age). Boys were the subject of 84.7% (160/189) of the queries, girls were the subject of 15.3% (29/189), and the gender of the child was not specified in the rest.

In 4.6% (9/195) of the queries, parents reported a family history of ASD. In 30.8% (60/195) of the queries, parents did not mention reporting their concern to a health care provider.

### Procedures

The content analysis of Yahoo Answers queries was conducted using the NVIVO software. Two types of content analysis processes were applied:

First, one type of content analysis was used to rate a child's risk of ASD as either low, medium, or high. To this end, a set of rules was devised for defining levels of ASD-risk from text. Medium-risk was defined by concerns related to one type of ASD-specific sign, general description of developmental delay, non-ASD–specific concern requiring evaluation and mentioning a risk factor for ASD such as a family member with ASD but no ASD-specific sign. For example:

My son is 19 months old & sometimes flaps his arms when excited or dancing, could he be autistic? he was born 3 months early & i do not know if this is normal behavior in a toddler or not...please help!

High-risk was defined as concerns related to at least two types of ASD-specific sign, 1 from the RRBI domain and another from the Social and Communication domains. High-risk rating also considered the severity of the described signs and urgency expressed by the parent:

My son is 2 1/2 years old and he can count to 15 and sing the whole abc song but he is not speaking with meaning asking me for things like juice and so. He also repeats long sentences from cartoons all day long but has nothing to do with what he is doing at that moment...

Low-risk was defined, by default, as queries that did not meet the above criteria, for example:

My child has a bent index finger on both hands. Sometimes it straightens out does this mean she has autism?

Then, 2 clinical experts in ASD separately rated the risk level of children described in 38 (19.49%) queries and reached kappa of .72 in their differentiation of at risk queries. Finally, ASD risk in the remaining queries was rated by 1 clinical expert.

Second, another process of content analysis was conducted to identify the types of warning signs noted by parents. This process, which involved deductive and inductive analysis methods, was conducted by a different clinical expert. The deductive method implied coding the warning signs according to domains and subdomains that match DSM-V criteria for ASD. An inductive method was used to identify concerning signs that did not correspond to the DSM-V diagnosing criteria such as describing cognitive impairment or language delay. The resulting taxonomy of the concerning signs mentioned in parents’ queries consisted of 12 domains and 72 subdomains (Multimedia Appendix 1). The first author and clinical expert coded 38 queries and obtained an inter-rater agreement as measured by significant kappa values between .54 and 1 across subdomains, with .71-1 values for the 12 domains. Due to challenges in agreeing on the number of manifestations pertaining to a single type of warning sign, each subdomain of sign (ie, the lowest hierarchy level within a subdomain) was coded for its presence in the query, regardless of the number of times that type was mentioned in the query (eg, “rocks, swings and sways body” is coded once for rocking). While coding, the clinical expert and the first author discussed coding dilemmas and refined the coding rules accordingly. To summarize, each query received an ASD global risk score and was coded for either presence or absence of each sign domain and its subdomains.

### Data Analysis

Data regarding the age and gender of the child were extracted using a combination of automated text analyses, followed by crowdsourcing rating to correct for errors. The sample with available age data was divided into 4 age groups: 0-2 (n=62), 2-3 (n=52), 3-6 (n=58), and ≥6 years (n=18).

Only signs with an occurrence of more than 5% were included in the analyses. Alpha level (Type 1 error) was corrected using Bonferroni for multiple comparisons, such that the threshold *P* value (.05) was divided by the number of comparisons. The sign domains differentiating the 3 ASD risk groups were analyzed using chi-square tests, given their dichotomous nature and Fisher’s exact tests for pairwise comparisons. The null hypothesis was that the distribution of sign domains across groups is equal. The association between the child’s gender and age group and the ASD-risk level was determined using chi-square tests. The length of a query differed significantly between ASD-risk groups (*F*_2,192_=10.93, *P*<.001; mean number of words associated with each risk level was as follows: low=132.57, medium=225.32, high=281.62). Bonferroni post-hoc tests indicated that the number of words was significantly (*P*<.05) higher in the high-risk group relative to the low-risk and medium-risk groups.

Data analysis was then conducted to assess whether children at risk of ASD could be detected from the queries using the text-coding method devised. The goal of this analysis was to differentiate between low-risk, medium-risk, and high-risk queries. To this end, each query was analyzed to determine (1) the number of times each word or word pair (bigram) appeared in the text, and, separately, (2) the warning signs, coded according to domains and subdomains, as previously explained.

Additional attributes analyzed included the child’s age and gender, the number of words in the query, as well as the length of the query, as measured by the number of characters in it. We trained a linear classifier [31] (with a least-squares criterion) and estimated its performance in terms of the receiver operating curve (ROC) using Leave-One-Out, that is, for each query *i*, we trained a classifier using all other queries, and tested on the *i*-th query [36].

## Results

**Figure 1:**
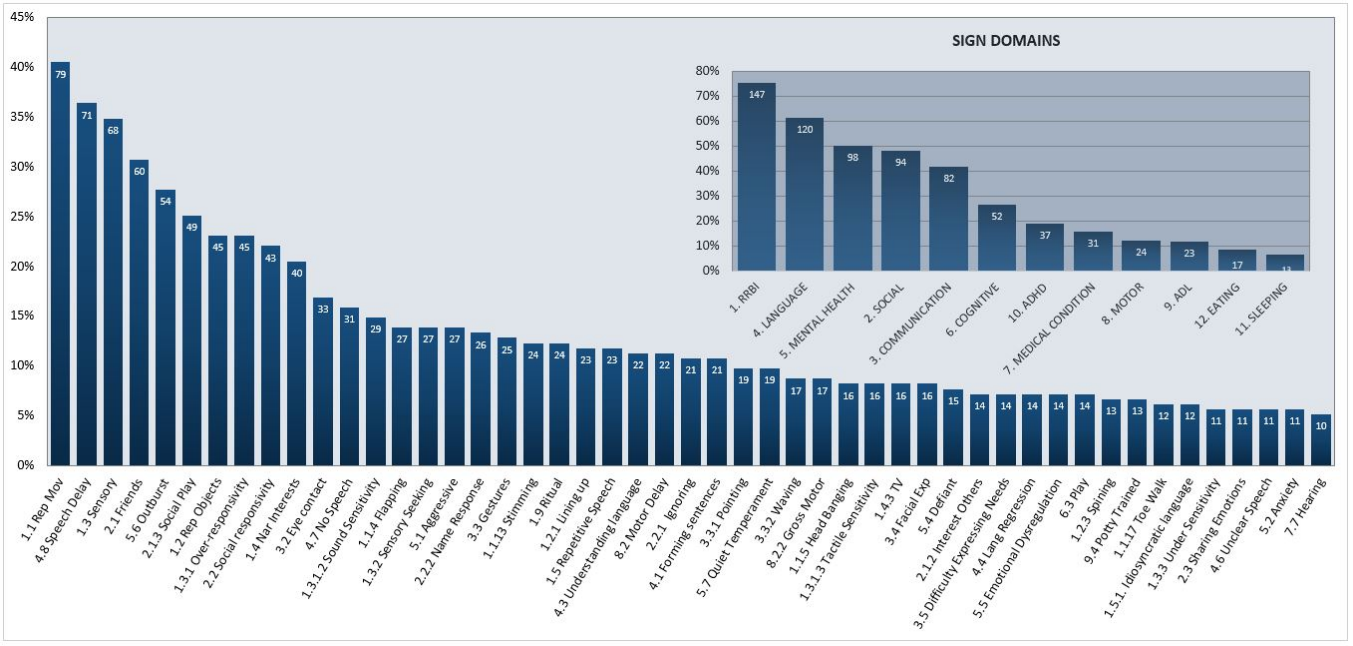
Percentage of domains and sub-domains of warning signs mentioned in ≥5% of queries. RRBI: repetitive and restricted behaviors and interests; ADHD: attention deficit hyperactivity disorder; ADL: activities of daily living

More than one third of the concerns were in ASD-specific domains: RRBI 75.4% (147/195), social 48.21% (94/195), and communication 42.05% (82/195); as well as non-ASD–specific domains: language 61.5% (120/195), emotional 50.3% (98/195), and cognitive 26.7% (52/195). Other sign domains that were mentioned in at least 5% of the queries were attention deficit hyperactivity disorder (ADHD) 18.97% (35/195), medical conditions 15.9% (31/195), motor 12.3% (24/195), activities of daily living (ADL) 11.8% (23/195), eating 8.7% (17/195), and sleeping problems 6.7% (13/195). The distribution of the types of warning signs mentioned in queries is presented in Figure 1. The 4 most prevalent types of signs were: repetitive movements (40.5% [79/195]), speech delay (36.4% [71/195]), sensory issues (34.9% [68/195]), and difficulties making friends (30.8% [60/195]).

The distribution of queries among the 3 ASD-risk levels, as determined by clinical experts, was: low-risk (n=35), medium-risk (n=60), and high-risk (n=100). The distribution of sign domains mentioned in queries is presented in Table 1 according to the level of ASD risk; results from chi-square tests comparing groups are also shown. Fisher’s exact pairwise comparisons reflected the significant difference between the high-risk and the other 2 groups in terms of ASD-specific sign domains. A surprising finding was the lack of significant difference in the types of ASD-specific concerns mentioned in low versus medium ASD-risk groups. There were significantly fewer (*P*<.001) language concerns in the low-risk group than in the other groups, which did not differ with regard to this domain. Exploratory analysis indicated that within each domain there were individual signs that contributed to the domain differences between the groups (Table 1).

**Table 1:** Distribution of domains of warning signs mentioned in queries presented according to ASD-risk levels.

Domain of signs	Risk group, n (%)			χ^2^	Differentiating subdomains^a^
	Low-risk (n=35)	Medium-risk (n=60)	High-risk (n=100)		
RRBI^b^	15 (42.9)^c^	37 (61.7)^c^	95 (95.0)^d^	46.78^d^	1.3 Sensory issues 1.3.1 Over-responsivity 1.3.1.3 Tactile sensitivity 1.2 Stereotyped or repetitive use of objects 1.4 Unusual and narrow interests 1.1.13 Stimming 1.5.1. Idiosyncratic language
Social	3 (8.6)^c^	18 (30.0)^c^	73 (73.0)^d^	54.61^d^	2.1 Difficulties in making friends, 2.1.3 Social play
Communication	9 (25.7)^c^	15 (25.0)^c^	58 (58.0)^d^	21.43^d^	
Language	8 (22.9)^c^	40 (66.7)^d^	72 (72.0)^d^	27.42^d^	
Emotional	14 (40.0)	29 (48.3	55 (55.0	2.46	
Cognitive^a^	3 (8.6)	13 (21.7)	36 (36.0)	11.08	
Medical conditions	5 (14.3)	11 (18.3)	15 (15.0)	0.40	
Motor^e^	1 (2.9)	6 (10.0)	17 (17.0)	5.23	
ADL^f^	2 (5.7)	7 (11.7)	14 (14.0)	1.71	
ADHD^g^	2 (5.7)	11 (18.3)	24 (24.0)	5.66	
Sleeping^e^	2 (5.7)	2 (3.3)	9 (9.0)	2.00	
Eating	3 (8.6)	3 (5.0)	11 (11.0)	1.70	

^a^*P* ≤.001.

^b^RRBI: repetitive and restricted behaviors and interests.

^c,d^Risk groups with different subscripts differed significantly in Fisher’s exact pairwise comparisons. For the significantly different domains, the signs differentiating risk groups were determined using chi-square tests with *P*<.001. Note that all hierarchies of signs with 5% occurrence and above were analyzed.

^e^Warning signs pertaining to these domains were observed in less than 5% of the queries.

^f^ADL: activities of daily living.

^g^ADHD: attention deficit hyperactivity disorder.

**Figure 2:**
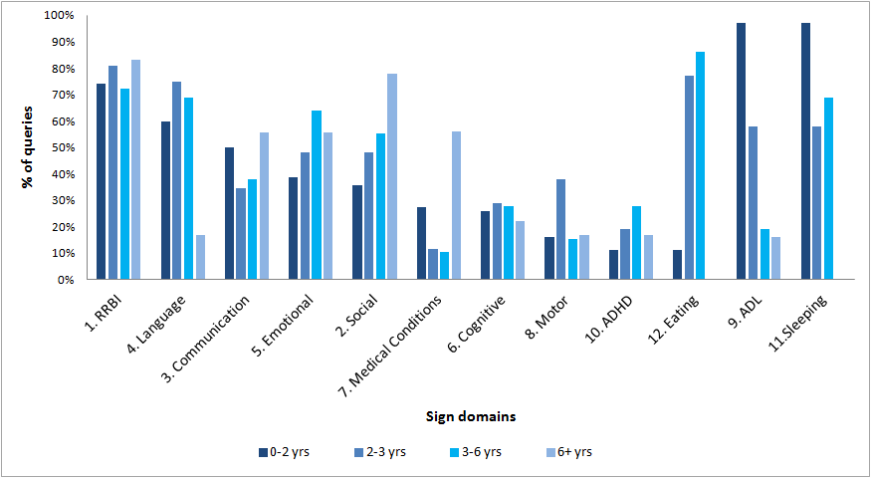
Percentage of sign domains mentioned in queries by age groups. RRBI: repetitive and restricted behaviors and interests; ADHD: attention deficit hyperactivity disorder; ADL: activities of daily living.

Looking at the association between the child’s age and level of risk indicated that the percentage of queries from each risk group did not differ between the 4 age groups (χ^2^_6_ =11.39, *P*=.08). There was a significant difference in the frequency of parents reporting language-related signs (see Figure 2 for pairwise comparison results between age groups using Fisher’s exact tests). Very few parents reported language signs in the oldest age group versus the other groups (16.7% [3/18] relative to 59.7% [37/62] to 75.0% [39/52] in the other age groups, χ^2^_3_=20.87, *P*<.001). Note that no significant difference was found in terms of the percentage of queries pertaining to boys versus girls in each risk group (eg, high-risk was 50% [80/160] and 58.6% [17/29], respectively, χ^2^_2_=6.17, *P*=.04).

**Figure 3:**
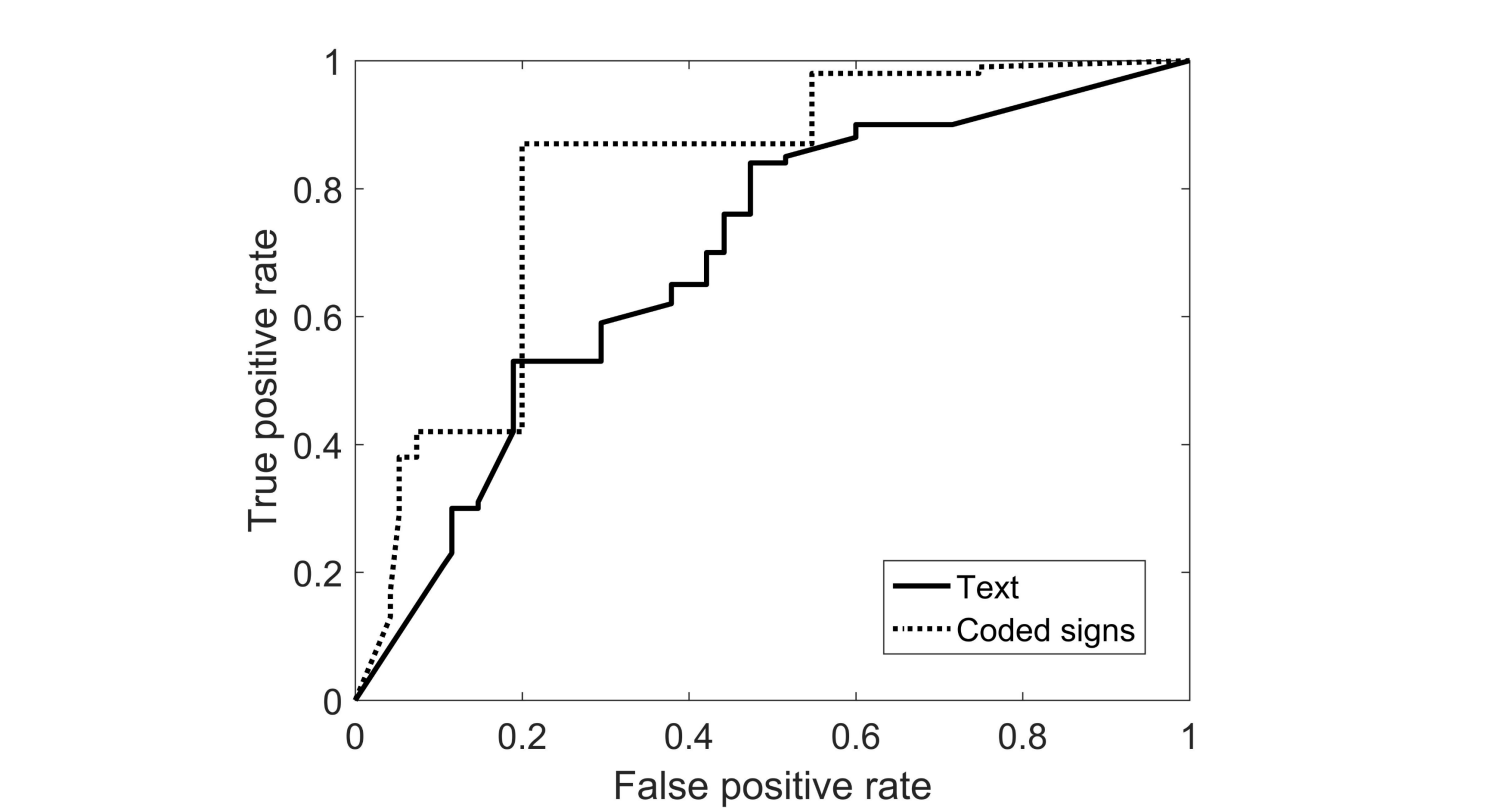
Receiver operating curve (ROC) plots predicting risk from text versus coded signs.

Next, we tested the efficacy of an automated, text-based ASD-risk estimator. When distinguishing high-risk queries from low- and medium-risk queries, the Area under the ROC curve (AUC) was found to be 0.67 (0.50-0.78). The AUC for this task using coded signs was 0.82 (0.80-0.86; see Figure 3). The latter result is significantly better than that obtained using the text alone (*P*=.002). Using both textual descriptions and coded signs reduced the AUC compared with using coded signs alone, probably because of the high dimensionality of the data, relative to the number of queries. Distinguishing low-risk from medium- and high-risk queries, the AUC using text was 0.54 and using signs was 0.84. Thus, text was a poor predictor for this task, compared with both signs and to the low- and medium-risk versus high-risk text classification.

Finally, we created a regression model to predict the actual risk score from the text and (separately) from signs. The Spearman correlation using text was .29 (*P*<.001), whereas the same using signs was .61 (*P*<.001). Thus, the actual risk score can also be deduced with higher accuracy from signs than from the text.

**Figure 4:**
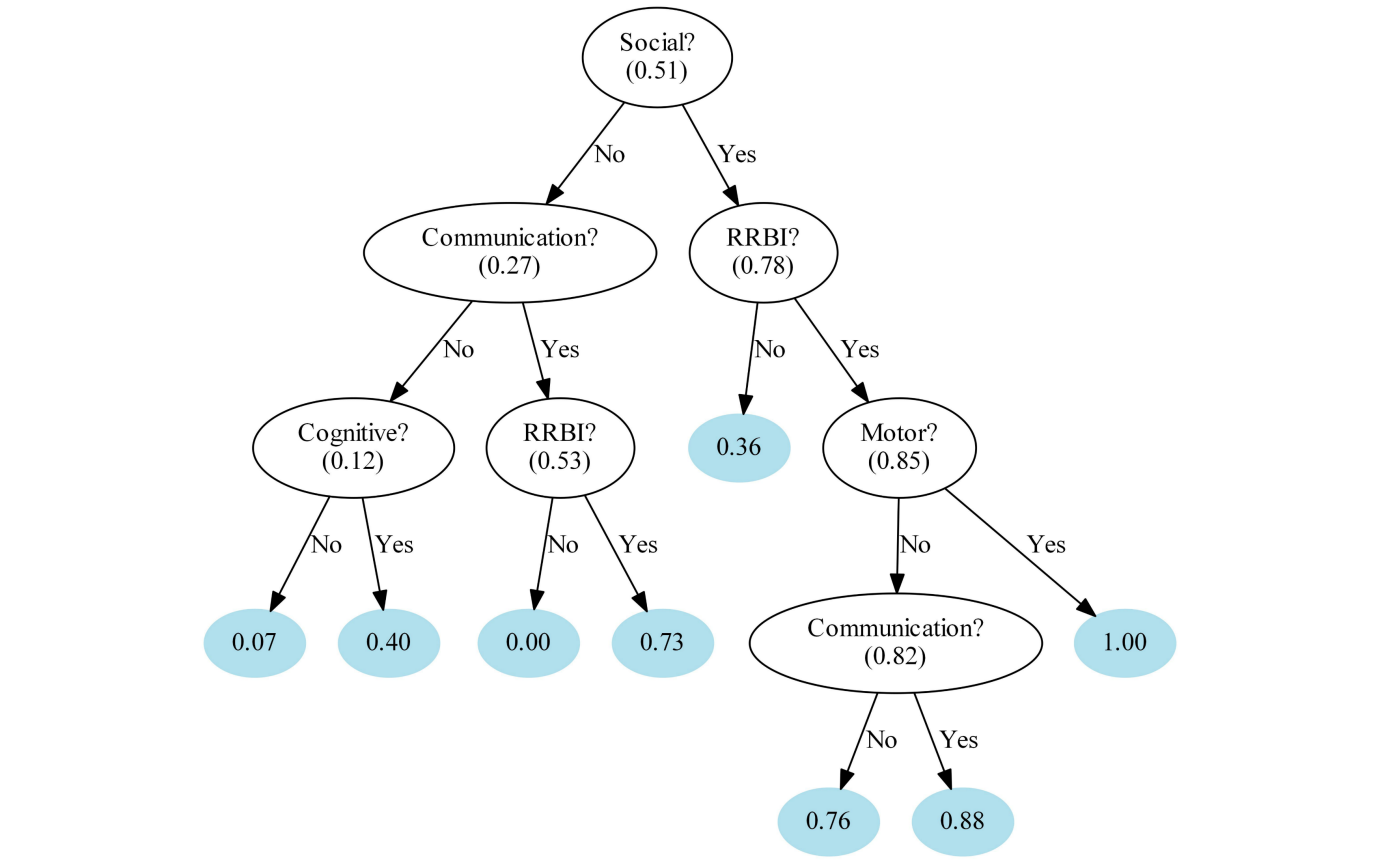
Decision tree classifier for distinguishing low-risk queries from medium- and high-risk queries. RRBI: repetitive and restricted behaviors and interests.

Figure 4 represents the decision-tree classifier for distinguishing low- and medium-risk from high-risk queries. Each node shows the coding variable used for decision and the fraction of high-risk queries at the node. The numbers at the end of the branch indicate the likelihood of high-risk for that branch. Social, RRBI, communication, cognitive, and motor concern domains entered the final model. Children who were mentioned to have a social problem had a 78% (73/94) chance of being at high-risk, compared with a 27% (27/101) chance in the rest of the sample. If the parent also reported an RRBI and motor delay, they had a 100% (13/13) chance of being in the high-risk group.

## Discussion

### Principal Findings

Our study examined the nature of online queries of parents who were concerned that their child might have ASD. The Internet offers parents a venue for expressing and verifying their concerns anonymously at any time and place. The analysis of these narratives highlights signs that alert parents, in the general public, of the possibility of their children having ASD. These concerns mirror parental developmental knowledge, awareness, and expectations, as well as levels of parenting anxiety. Most of the Yahoo Answers queries were judged by clinical experts as reflecting high-risk for ASD or as medium-risk, validating parents’ concerns. Differentiating ASD early in life is important, so that the child with ASD can gain the most from targeted interventions [37]. Parental concerns expressed online offer a new method for facilitating earlier screening and referral for evaluation. In the long run, an online tool which provides a gross estimate of ASD risk based on textual descriptions of warning signs and guided questions could prompt parents to approach a professional. Such a tool can harness social media to support worried parents and minimize the risk of acting on nonprofessional advice or disregarding worries.

### Comparison With Prior Work

Our findings that parents with mostly no family history of ASD (95.4% [186/195]) associated a broad range of ASD-specific signs with the disorder were encouraging. The prevailing signs that parents found worrisome were within the DSM-V [12] ASD core domain of RRBI, followed by the social-communication domain. Nevertheless, more than a third of the parents sampled, expressed concerns related to domains of language, cognitive, and emotional, which according to the DSM-V are non-ASD–specific; however, they are highly prevalent in ASD. Other non-ASD–specific concerns mentioned in some of the queries related to motor development, ADL, and medical conditions. Interestingly, concerns related to motor problems entered as a meaningful domain in the decision-tree classifier. The mix of concerns from ASD and non-ASD–specific domains is consistent with previous studies documenting the types of first parental concerns of children later diagnosed with ASD [9,10,15].

The average child age at which an online concern was raised was 38.03 months, close to the average age of ASD diagnosis [38,39]. Nonetheless, this age is greater than the average age of the first concerns that was reported in ASD research, which is 10-18 months [5,9,19,21,24]. The majority of queries described sons, although analysis of queries did not reveal different types of signs for sons versus daughters, and the rate of ASD risk was not significantly higher for boys than for girls. Comparisons between age groups showed that language concerns differed in prevalence across age groups. Language problems were mentioned most frequently in queries regarding children 0-3-year-old and less in the oldest group. It is likely that parents of a nonverbal 7-year-old have reached the stage beyond suspicion and hence would be less likely to query this. Note that the peak of emotional concerns was in the 3-6 years old age group, particularly descriptions of outbursts or extreme shyness. Social concerns increased with highest prevalence in the older group. These differences between age groups can be explained in the light of the changes in developmental expectations of parents from children at different ages.

At the extremes, there were parents who raised concerns too early to determine risk, for example:

My 8-week-old son has yet to flash his first true smile. I know that all babies develop differently, but i am worried about autism...

At the other end of the spectrum, there were parents of older children who were either questioning a non-ASD diagnosis their child received or were never evaluated but always felt something was different, for example:

(regarding an 18-year-old child) we always knew he was different, he displays some—but not all—of the common symptoms, but we just put it down to him being different and an introvert...apart for the virtual world of computer games he is turning into a hermit...and has no social skills.

When designing an online screening tool, the age of the child must be considered and, based on the findings, the need to continue monitoring such a child is warranted.

Although there were differences in rates of RRBI concerns across risk groups, they were mentioned in at least 75.4% [147/195] of the queries. The RRBI domain included the largest number of hierarchy levels of subdomain coding as well as individual signs (the subdomains are: repetitive movements, stereotypes and repetitive use of objects, sensory issues, unusual and narrow interests, repetitive speech, eating, difficulty with change, rigid thinking, and rituals). This reflects the diversity in types of symptoms described in the DSM-V criteria. The most prevalent RRBIs mentioned were repetitive movements, repetitive use of objects, and sensory abnormalities. The RRBIs characterizing queries of medium- or high-risk were repetitive speech, sensory issues (particularly tactile over-reactivity), unusual use of objects, repetitive speech (particularly idiosyncratic language), and repetitive interest. Interestingly, research shows that RRBIs are not the most prevalent first concerns of parents of children later diagnosed with ASD [18,22]. It may be the most reported domain in online queries, as parent’s attention is more easily drawn to atypical behaviors or socially inappropriate behaviors, compared with their ability to recognize a delay in attaining a milestone. Signs within this domain can be intense and can interfere with play and participation, and thus are noticeable. The presence of some RRBIs in typical development (eg, head banging, noise sensitivity) presents a further challenge for relying on this domain to verify risk status. There is a need to increase parental awareness of the typical manifestations of RRBI during the first 3 years of life, to help parents understand when such concerns may be warranted. Online concerns reflect a parent’s call for help regardless of whether a child has ASD. Therefore, there is clearly a need for interactive parenting education materials aimed at interpreting and coping with RRBIs.

Our results indicated that it is possible to predict risk from the text using machine learning methods once the text is classified into sign domains, whereas using text alone provided insufficient information (at least in our corpus) for accurate identification of children at risk. Developing an ASD-specific flowchart into which parents could insert their narratives related to certain types of concerns may provide a basis for a more accurate prediction of ASD risk from text. An automated screening tool in online forums will benefit from starting with a social concern question, and if not present then ask about communication while if present ask about the presence of RRBIs. While ASD specific questions will need to dominate such a tool, probing about cognitive and motor markers, which are not ASD-specific, is also important. Results from the decision tree indicate that the combination of signs from the social, RRBI, and motor domains predicted the highest likelihood of ASD risk from the coded text. This is in line with the evidence showing that parental concerns pertaining to a combination of several domains predicted an ASD diagnosis [3]. The prediction of high risk from text or text combined with coded signs was better for the high ASD risk group alone rather than predicting for both medium- and high-risk groups. Future research relying on a larger corpus could robustly test different combinations of concerns in predicting ASD risk. As parents seek first-level support online, developing Internet- and mobile-health tools to automate ASD screening relying on the decision-tree classification described in this study may reduce screening time, increase response to screening, and increase accuracy.

The distribution of the types of signs mentioned in online queries did not fully correspond to the signs most commonly reported in previous studies. For instance, in an ASD study, parental language and communication concerns were found to be the most prevalent early concerns, followed by social, RRBI, medical, and emotional domains [15]. Communication signs were highly prevalent among parental concerns noted in other studies as well; however, they did not consistently differentiate those later diagnosed with ASD [3,9,10,17,19,20,22]. These differences in the most common ASD concerns of parents may be explained by the fact that previous studies relied on retrospective reports and samples characterized by high genetic risk for ASD [3,15,19,22,25], as opposed to a sample with little familiarity with ASD symptomatology and a lower likelihood for the child to have ASD.

### Limitations

The limitation of the current exploratory study is the lack of clinical testing for the actual ASD-risk status of the child as opposed to other neurodevelopmental disorders. The next step would be to study the external validity of risk status using standardized developmental measures, develop a structured format for parents to enter their concerns and, test our algorithm for predicting risk from text with a new corpus. Extracting signs from text has its own limitations, as in some cases it requires the clinical inference of meaning, as opposed to the ability to probe a parent and thus extract the meaning behind the concern. We attempted to minimize such bias by developing coding rules and testing coding reliability. The anonymous nature of online queries makes online parental concerns a unique resource that offers an authentic snapshot of parental ASD-related concerns, unaffected by issues of social desirability or other emotional biases.

### Conclusions

Early parental concerns constitute a valuable component of early childhood screening. There is accumulating evidence that early parental concerns regarding specific ASD markers are associated with a higher likelihood of an eventual ASD diagnosis [3,15,40]. Our study is the first to investigate online queries describing the types of signs that lead parents to suspect ASD in their child. The fact that the clinical experts found that the majority of queries corresponded to either medium- or high-risk for ASD validated the need to facilitate the parents’ earlier consultation with a professional. We showed the potential of utilizing machine learning methods for ASD screening based on parental concerns. Findings also highlight the need for designing parent-education tools regarding behaviors that are age appropriate, particularly those pertaining to the RRBI domain. Finally, it is important to empower parents’ confidence in their concerns and increase their awareness of the disadvantages of relying solely on an online community for determining ASD-risk status. This study’s findings support the call for health care providers to closely listen to parental ASD-related concerns, as recommended by screening guidelines [13]. Results also demonstrate the need for Internet-based screening systems that utilize parents’ narratives combined with a hierarchical screening questioning. Worried parents approach online communities, comprised mostly lay person answerers, to obtain opinions regarding their child’s likelihood of having an ASD diagnosis. A more efficient mechanism for supporting worried parents online is important for prompting a clinical evaluation when needed and reducing parental anxiety.

## Multimedia Appendix 1

Taxonomy domains and sub-domains of warning signs coded from Yahoo queries.

## Abbreviations

ADHD: attention deficit hyperactivity disorder
ADL: activities of daily living
ASD: autism spectrum disorders
AUC: area under the curve
DSM-V: Diagnostic and Statistical Manual of Mental Disorders
M-CHAT-R/F: Modified Checklist for Autism in Toddlers-Revised with Follow-Up
MTV: music television
ROC: receiver operating curve
RRBI: repetitive and restricted behaviors and interests
