# Supplementary material for: Online Concerns of Parents Suspecting Autism Spectrum Disorder in Their Child: Content Analysis of Signs and Automated Prediction of Risk
Source: J Med Internet Res. 2016 Nov 22;18(11):e300. doi: 10.2196/jmir.5439 (PMC5141337; doi:10.2196/jmir.5439)
Supplement: Supplementary file 1 [file jmir_v18i11e300_app1.pdf]

Appendix A. *Taxonomy Domains and Sub-Domains of Warning Signs Coded from Yahoo Queries*

Domain Codes	Sub-domain Codes	Examples from Queries
1. RRBI	1.1. Repetitive movements:<br>1.1.1. Back arching<br>1.1.2. Clenched fists<br>1.1.3. Constantly masturbating<br>1.1.4. Hand flapping<br>1.1.5. Head banging<br>1.1.6. Head movements<br>1.1.7. Head rubbing<br>1.1.8. Jumping<br>1.1.9. Pacing<br>1.1.10. Pinching his neck<br>1.1.11. Rocking<br>1.1.12. Running<br>1.1.13. Stimming<br>1.1.14. Teeth grinding<br>1.1.15. Tongue chewing<br>1.1.16. Touching ears	"He flaps his hands. He suddenly starts jumping back and forth for hours. He makes weird noises. he walks back and forth"<br>"Seems completely obsessed with cars, but that's really all he plays with he shows little interest in other toys"

Domain Codes	Sub-domain Codes	Examples from Queries
	1.1.17. Walking on toes	
	1.1.18. Weird facial expressions	
	1.1.19. Wiggling toes	
	1.2. Stereotyped or repetitive use of objects:	
	1.2.1. Lining up objects	
	1.2.2. Pushing buttons	
	1.2.3. Spinning	
	1.2.4. Toys	
	1.3. Sensory issues:	
	1.3.1. Over-responsivity:	
	1.3.1.1. Low pain threshold	
	1.3.1.2. Sound sensitivity	
	1.3.1.3. Tactile sensitivity:	
	1.3.1.3.1.Doesn't like to be cuddled	
	1.3.1.3.2.Sensitive to hair brushing	
	1.3.1.3.3.Sensitive to nail cutting	
	1.3.1.3.4.Sensitivity to clothes	
	1.3.1.3.5.Sensitivity to tooth brushing	
	1.3.1.4. Vestibular sensitivity	

Domain Codes	Sub-domain Codes	Examples from Queries
	1.3.1.5. Vision sensitivity:	
	1.3.1.5.1. Sensitivity to lights	
	1.3.1.5.2. Sensitivity to sunlight	
	1.3.2. Seeking:	
	1.3.2.1. Likes to smell everything	
	1.3.2.2. Loves different textures	
	1.3.2.3. Loves loud noises	
	1.3.2.4. No sense of danger	
	1.3.2.5. Proprioceptive seeking	
	1.3.2.6. Vestibular seeking	
	1.3.2.7. Vision:	
	1.3.2.7.1. Eye rolling	
	1.3.2.7.2. Moves objects in front of eyes	
	1.3.2.7.3. Sideways glances	
	1.3.2.7.4. Staring	
	1.3.3. Under sensitivity:	
	1.3.3.1. No sense of pain	
	1.4. Unusual and narrow interests:	
	1.4.1. Fascination with feces	

Domain Codes	Sub-domain Codes	Examples from Queries
	1.4.2. Lego<br>1.4.3. TV<br>1.4.4. Vehicles<br>1.5. Repetitive speech:<br>1.5.1. Idiosyncratic language<br>1.5.2. Gibberish<br>1.5.3. Makes weird noises<br>1.5.4. Unusually formal language<br>1.6. Difficulty with change<br>1.7. Rigid thinking<br>1.8. Ritual	
2. Social	2.1 Difficulties in making friends:<br>2.1.1 Difficulties understanding social contexts<br>2.1.2 No interest in others<br>2.1.3 Social play<br>2.2 Social responsivity:<br>2.2.1 Ignoring<br>2.2.2 Responds to name<br>2.2.3 Talks to himself	"Mostly he is affected socially he is extremely shy. He does not play with other children at parties, preschool only watches them. at home he is interactive with siblings though"

Domain Codes	Sub-domain Codes	Examples from Queries
	2.3 Reduced sharing of emotions:<br>2.3.1 Not affectionate<br>2.3.2 Selective social response<br>2.4 Social anxiety<br>2.5 Social delay	
3. Communication	3.1 Difficulty with reciprocal conversation<br>3.2 Eye contact<br>3.2.1 Poor eye contact<br>3.3 Communicative gestures:<br>3.3.1 Pointing<br>3.3.2 Waving<br>3.3.3 Clapping<br>3.4 Problems with facial expression:<br>3.4.1 Smiling<br>3.4.2 Vacant look<br>3.5 Difficulty expressing needs<br>3.6 Lack of joint attention<br>3.7 Lack of imitation<br>3.8 Speech prosody:	“Eye contact is pretty rare”;<br>“She is not waving, not clapping, not pointing”<br>“His main problems are… not understanding what we are pointing at or trying to get him to look at”

Domain Codes	Sub-domain Codes	Examples from Queries
	3.8.1 Speaks quietly<br>3.8.2 Talks loudly<br>3.8.3 Talks slowly<br>3.9 Physical requests<br>3.10 Understand Non-verbal	
4. Language	4.1 Forming sentences<br>4.2 Initiating words<br>4.4 Regression<br>4.5 Speech apraxia<br>4.6 Unclear speech<br>4.7 No speech<br>4.8 Speech delay	"She will repeat a word after you say it, but really is not trying to communicate or make sentences"<br>"My baby boy 20 months old has lost his words"
5. Emotional	5.1 Aggressive and violent:<br>5.1.1 Animal cruelty<br>5.1.2 Bites<br>5.1.3 Hits himself<br>5.1.4 Pulls hair<br>5.2 Anxiety:<br>5.2.1 Divorce trauma	"Gets very mad to where he will hit himself"<br>"He has panic attacks"<br>"He throws severe tantrums; sometimes I don't even know why he is so upset"

Domain Codes	Sub-domain Codes	Examples from Queries
	5.2.2 Panic attacks	
	5.2.3 Phobias	
	5.3 Attachment	
	5.4 Defiant:	
	5.4.1 Stubborn	
	5.4.2 Lying	
	5.4.3 Not following orders	
	5.5 Emotional dysregulation	
	5.6 Outburst and tantrums	
	5.7 Quiet temperament	
	5.8 Wetting	
6. Cognitive problems	6.1 Following instructions	"When I teach him something he forgets for example, he forgot what is a cup"
	6.2 Attention and hyperactivity	
	6.3 Play problems:	
	6.3.1 Functional play	
	6.3.2 Symbolic play	
	6.4 Cognitive delay	
	6.5 Learning difficulties:	
	6.5.1 Arithmetic	

Domain Codes	Sub-domain Codes	Examples from Queries
	6.5.2 Colors and shapes	
	6.5.3 Literacy	
	6.5.4 Hyperlexia	
	6.5.5 Language comprehension	
7. Medical condition [a]	7.1 Allergy	
	7.2 Apraxia and Dyspraxia	
	7.3 Breathing problems	
	7.4 Cerebral palsy	
	7.5 Digestive system:	
	7.5.1 Constipation	
	7.5.2 Diarrhea	
	7.5.3 Gas pain	
	7.5.4 Green bowel	
	7.5.5 Irritable bowel	
	7.5.6 Vomit	
	7.6 Failure to thrive	
	7.7 Hearing:	
	7.7.1 Cochlear implant	
	7.7.2 Deafness	

Domain Codes	Sub-domain Codes	Examples from Queries
	7.7.3 Ear infections	
	7.7.4 Earwax problems	
	7.7.5 Fluid in the ears, tubes	
	7.8 High lead level	
	7.9 Jaundice	
	7.10 Low muscle tone	
	7.11 Emotional diagnoses:	
	7.11.1 OCD	
	7.11.2 ODD	
	7.12 Physical features:	
	7.12.1 Dysmorphic facial features	
	7.12.2 Eyes far apart	
	7.12.3 Flat nose	
	7.12.4 Left handed	
	7.12.5 Bent index finger	
	7.13 Diagnosed language impairment	
	7.14 Pregnancy problems	
	7.15 Preterm birth	
	7.16 Retardation	
	7.17 Seizures	
	7.18 Sensory processing disorder	

Domain Codes	Sub-domain Codes	Examples from Queries
	7.19 Tongue-tied<br>7.20 Tourette<br>7.21 Vision:<br>7.21.1 Blindness<br>7.21.2 Lazy eye<br>7.21.3 Not tracking objects<br>7.22 Illness following vaccination	
8. Motor	8.1 Motor play<br>8.2 Motor delay<br>8.2.1 Fine motor<br>8.2.2 Gross motor:<br>8.2.2.1 Holding head up<br>8.2.2.2 Jumping<br>8.2.2.3 Riding a bike<br>8.2.2.4 Crawling<br>8.2.2.5 Sitting<br>8.2.2.6 Walking	"He does not write very well at all. I have to work with him a lot to hold the crayon right. He does draw little stick figures and shapes but letters are very hard for him"<br>"He does not jump, pedal a bike<br>"He did not sit till 9m with cushion, sat alone at 12m, never crawled, walked at 12.5m established walking at two"
9. ADL	9.1 Eats or drinks independently<br>9.2 Gets dressed	"Clothes can appear inside out and on trouser and shorts will be inside out with the tag facing the outside"

Domain Codes	Sub-domain Codes	Examples from Queries
	9.3 Independent	"He cannot bathe or feed himself".
	9.4 Potty trained	
10. Sleeping		
11. ADHD		
12. Eating		

Note. All codes reflect a concern/difficulty within that area. [a] This includes diagnosed conditions.
